# Case of Utero-Vaginal Rupture

**Published:** 1841-04-24

**Authors:** James F. Wilson

**Affiliations:** Wilmington, Del.


### ORIGINAL COMMUNICATIONS.

*Case of Utero-Vaginal Rupture.* By James F. Wilson, M. D., of Wilmington, Del.

On Saturday, the 13th of March last, I was called to see C. D., aged 26, in labour with her third child. She had been complaining during the whole of the day before, but her pains were so slight that she did not take to her bed until a few hours before I saw her; which was about eleven o'clock on the morning of the 13th. Her pains at the time were not very severe.

On making an examination per vaginam, the os uteri was found to be but partially dilated, although it was very easily dilatable. As the membranes protruded very much, they were ruptured. The pains, however, did not increase; and as the os uteri was easily dilated, and the external parts disposed to yield, I gave her ten grains of the Secale Cornutum, which evidently increased the force of her pains. These effects soon passed away; the dose was repeated, and shortly after she had a few very severe pains. From this time the action of the uterus gradually declined, until about four o'clock, when it had almost entirely ceased. I now called on my friend Dr. Porter, and requested him to see the patient with me. We found the os uteri less dilatable than it was in the morning, and it was the opinion of both Dr. P. and myself, that the case demanded bleeding. Accordingly a vein was opened, and about eight ounces of blood taken, when we found the pulse becoming weak and frequent. Four hours after this (half past nine, P. M.) the os uteri was more dilated, but the pains were still weak and inefficient. We ordered an anodyne and left her, requesting her attendant to call us if she thought our presence would be required before we returned.

Sunday, nine o'clock, A. M. Passed a very restless night, having complained much of pains, but few of which the patient thought efficient. At this time she was almost entirely without pain. Examination per vaginam showed that the os uteri was fully dilated, and that the child had advanced from three-fourths of an inch to an inch beyond the situation it occupied at the last examination. It was now very evident that the breech was the presenting part, the spine of the child corresponding to the symphysis pubis, and the feet to the sacrum. Several ineffectual attempts were made to bring down the feet by the hand. Ergot was again administered, but did not produce the least effect.

Early in the afternoon we requested that Dr. Askew should see the patient with us; and in consultation it was agreed that the safety of the woman demanded that she should be delivered as soon as possible, and by the aid of instruments. Accordingly, Dr. Askew proceeded to bring down the feet by means of the blunt hook. This was very difficult to accomplish, and required a great degree of force. The right foot was first brought down, and shortly after the left; the body was soon expelled, and the arms brought down. The vertex now offered to the symphysis pubis and the forehead to the sacrum.

The patient was by this time so much exhausted that it was thought necessary to resort to the use of stimulants, and to leave her as just described till she should revive. At six o'clock, P. M., she was still very weak. The head could not be delivered by the forceps, consequently it was brought away by the crotchet, which was placed in the left temporal bone, directly over the mastoid process. The delivery of the head was extremely difficult. There was not the least subsidence of the abdominal tumor, until the delivery was nearly completed.

There was no very great loss of blood during the delivery; the womb contracted, the placenta was soon brought away and the tampon applied.

The patient was now moved to another part of the bed, when she was found to be almost pulseless. The use of stimulants was commenced immediately, and by ten o'clock there was considerable arterial action; the woman was easy and comfortable.

Monday 15th, 9 o'clock, A. M. We found her quite cheerful; complains of nothing. Rested very well last night, having complained of pain but twice; but was troubled more frequently with cramp in her legs. Pulse full and soft.

5 o'clock, P. M. Delirium, with very weak and frequent pulse. She continued to sink, and died about eight o'clock.

*Autopsy*, eighteen hours after death. The examination was made in the presence of several medical gentlemen. The abdomen was enormously distended. The fundus of the uterus was about half way between the pubis and the umbilicus. On making an incision through the linea alba, a great quantity of very fœtid gas escaped. In the lower part of the abdomen the peritoneum over the uterus was considerably injected. The redness was in patches of from two to three lines in circumference, with intermediate portions of this membrane in an uninjected state. There was no effusion of lymph. In the cavity of the peritoneum there were considerable effusion and coagula of blood. The most important appearances found on dissection were a deformity of the pelvis and a laceration of the uterus. The projection of the sacrum reduced the antero posterior diameter of the superior strait to three inches and a quarter. There was a transverse rupture of the left side of the neck of the womb, extending partly into the vagina; the laceration was about two inches and a half in length. That part of the anterior surface of the womb corresponding to the symphysis, presented somewhat the appearance of a bruise, and was easily torn by the finger. At this place, beneath the peritoneal membrane, there was an effusion of dark blood-like matter of several inches in extent; the womb, around the rupture, presented the same appearance. The thickness of the womb about the fundus was from an inch to an inch and a half; near the rupture about one inch.

The interior of the uterus was covered by patches of partly coagulated blood; when this was removed the inner face of the uterus was rough and presented a red appearance.

Before concluding the relation of this case, I would merely remark, that her preceding labours were of that slow and tedious class which require the use of ergot; and it is not improbable that, in one of them at least, instruments were used: certain it is, that in both cases the child was still-born.

It would be interesting to determine at what time the laceration occurred. We know that it may take place in any stage of labour, and from a variety of causes—but as those alarming symptoms which indicate this dreadful accident, such as violent screaming at the time of its occurrence, followed by vomiting of dark coloured fluid, ghastly countenance, oppressed breathing, fainting, &c., were all absent, I am at a loss to come to any satisfactory conclusion, with regard to the time at which the accident happened.

How are we to account for the apparent convalescence of the patient on the morning after her delivery? With restored strength, she had not the slightest appearance of fever; on the contrary, every circumstance of the case indicated a speedy return of her usual health.

